# Necrostatin-1 treatment inhibits osteocyte necroptosis and trabecular deterioration in ovariectomized rats

**DOI:** 10.1038/srep33803

**Published:** 2016-10-05

**Authors:** Hongwang Cui, Yongjun Zhu, Qiming Yang, Weikang Zhao, Shiyang Zhang, Ao Zhou, Dianming Jiang

**Affiliations:** 1 Department of Orthopedics, The First Affiliated Hospital of Chongqing Medical University, Chongqing, China; 2 Department of Nephrology, The First Affiliated Hospital of Chongqing Medical University, Chongqing, China

## Abstract

Estrogen (E2) deficiency has been associated with accelerated osteocyte apoptosis. Our previous study showed necroptosis accelerated the loss of osteocytes in E2 deficiency-induced osteoporosis in rats in addition to apoptosis, but the mechanism involved remains. Necroptosis is a caspase-independent form of programmed cell death. In the necroptosis pathway, receptor interaction proteins 1 and 3 (RIP1/3) play vital roles. Necrostatin-1 (Nec-1) has been confirmed to be a specific inhibitor of necroptosis. However, the effect of Nec-1 on postmenopausal osteoporosis remains ambiguous. The aim of this study was to investigate the effect of Nec-1 on osteocytes in ovariectomized (OVX) rats. We found that an increased number of necroptotic osteocytes was related to the production of tumor necrosis factor-alpha (TNF-α) in OVX rats. Treatment with Nec-1 significantly decreased RIP1 and RIP3 expression in OVX rats and inhibited osteocyte necroptosis induced by TNF-α *in vitro*. Both E2 and Nec-1 treatment markedly ameliorated trabecular bone deterioration. Nec-1 also significantly elevated the levels of bone formation markers and decreased bone resorption markers. These data suggest that the role of Nec-1 on alleviating bone loss might be associated with Nec-1 restraining TNF-α-induced osteocyte necroptosis in rats with E2 deficiency-induced osteoporosis. This process may represent a novel therapeutic strategy for the treatment of postmenopausal osteoporosis.

Postmenopausal osteoporosis (PMOP) is one of the primary forms of osteoporosis and occurs after estrogen (E2) with drawal in women. Typically, the dual processes of bone resorption and formation are tightly coupled to maintain a steady state of bone homeostasis^1^. With PMOP, certain amounts of bone loss occur in postmenopausal women, tipping the balance toward increased bone resorption coupled with decreased formation. However, the exact mechanism underlying PMOP remains poorly understood. Although osteoblast and osteocyte apoptosis have been confirmed to be important in PMOP^2^, there are still a number of questions that deserve further attention. Apoptosis is a highly regulated process involving the caspase family of cysteine proteases. In addition to apoptosis, there may be numerous other mechanisms that regulate cell death and can lead too steocyte loss. In our previous study, we found that ovariectomy-induced E2 loss in the femurs of female rats resulted in an increased proportion of osteocytes undergoing necroptosis, in addition to apoptosis^3^.

Several studies have demonstrated estrogen deficiency increases the production of tumor necrosis factor-alpha (TNF-α) in ovariectomized (OVX) rats^4^,^5^. Therefore, TNF-α has been recognized as a potent stimulator of bone resorption^6^. However, the role of TNF-α in determining necroptotic osteocytes remain unsolved.

Necroptosis is a form of caspase-independent programmed cell death associated with morphological changes similar to those that occur in necrosis. Receptor-interacting proteins 1 and 3 (RIP1/3) both play vital roles in the necroptotic pathway^7^,^8^,^9^. Necrostatin-1 (Nec-1, an inhibitor of RIP1 activity) has been confirmed to be a specific inhibitor of necroptosis^10^. Nec-1 functions as a cellular protectant in adult rodent models of chronic kidney disease^11^, ischemic brain injury^12^ and myocardial infarction^13^. A previous study showed that Nec-1 enhanced bone formation under glucocorticoid-induced osteoporosis in rats^14^. However, the effect of Nec-1 on E2 deficiency-induced osteoporosis remains ambiguous. Therefore, the objective of this study was to test the effect of Nec-1 on bone metabolism in an OVX rat model.

Using a combination of techniques, including the examination of cell morphology, molecular biology, and histomorphometric investigations, the current study demonstrates that necroptotic osteocytes might be induced by TNF-α *in vivo* and *vitro*. We investigated that Nec-1 can prevent necroptotic osteocytes, mediated by RIP1/3, and inhibit osteoporosis progression in OVX rats. We further identify RIP1 as a potential therapeutic target to prevent bone loss in postmenopausal women.

## Results

### Effect of Nec-1 on necroptosis-related proteins in OVX rats

**Figure 1:**
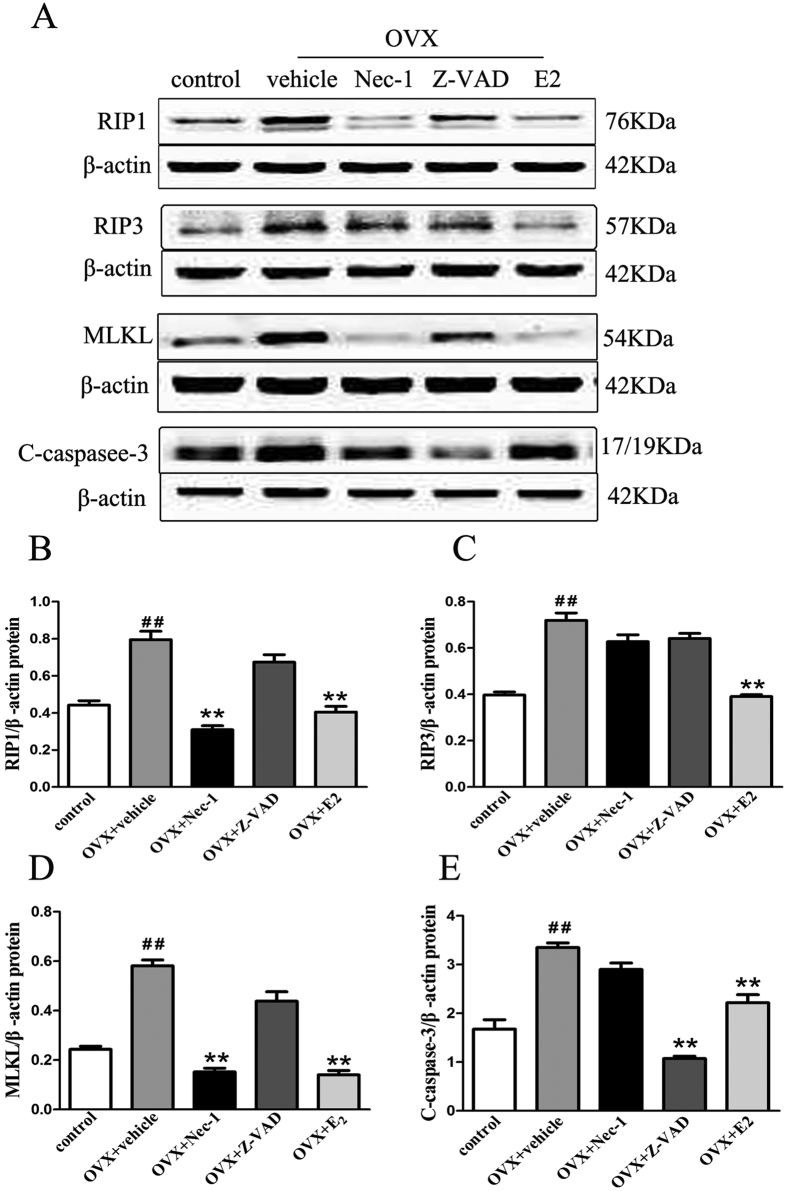
Western blotting analysis of RIP1, RIP3, MLKL and cleaved caspase-3 protein levels in the bone tissue of OVX rats. Rats underwent OVX surgery, recovered for 4 weeks, and were injected intraperitoneally with either Nec-1 (1.65 mg/kg/d), Z-VAD (1.0 mg/kg/d), E2 (10 μg/kg/day) or an equal volume of 10% DSMO(vehicle) and were maintained for 4 more weeks prior to experimental analyses. The control group was treated with the vehicle solution. At 8 weeks post-surgery, the bone lysates were subjected to immunoblotting with specific antibodies. Immunoblot signals were quantified and normalized to β-actin. (**A**) Representative blots are shown. Western blotting analyses for RIP1 (**B**), RIP3 (**C**), MLKL (**D**) and cleaved caspase-3 (**E**) protein expression in the bones of rats in each treatment group are shown. The results are shown as the means ± S.E.M for three independent measurements. N = 6 rats per group, ^##^p < 0.01 compared with the control group, **p < 0.01 compared with the vehicle group. OVX: ovariectomized.

RIP1 and RIP3 both play important roles in the necroptosis pathway^8^. RIP3 is a key regulator of RIP1 kinase activation^15^, and its expression level has been shown to correlate with necroptosis^16^. New studies have found that the mixed lineage kinase domain-like protein (MLKL), a key signaling molecule downstream of RIP3, can form a complex with RIP1 and RIP3 to trigger necroptosis^17^. Therefore, we assessed RIP1 (Fig. 1A,B), RIP3 (Fig. 1A,C), MLKL (Fig. 1A,D) and caspase-3(Fig. 1A,E) protein expression in the proximal femurs of rats 8 weeks after OVX by western blotting. RIP1, RIP3, MLKL and caspase-3 protein expression levels were at low levels in the control group, increasing sharply in the vehicle group (p < 0.01). Nec-1 significantly inhibited RIP1 and MLKL expression but had no effect on RIP3 or caspase-3. Treatment with Z-VAD (Z-VAD-fmk) significantly inhibited caspase-3 protein levels but had no effect on RIP1, RIP3 or MLKL. Compared with the vehicle rats, E2 significantly inhibited RIP1, RIP3, MLK and caspase-3 protein expression (p < 0.01).

### Nec-1 prevents E2 deficiency-induced osteocyte necroptosis in OVX rats

**Figure 2:**
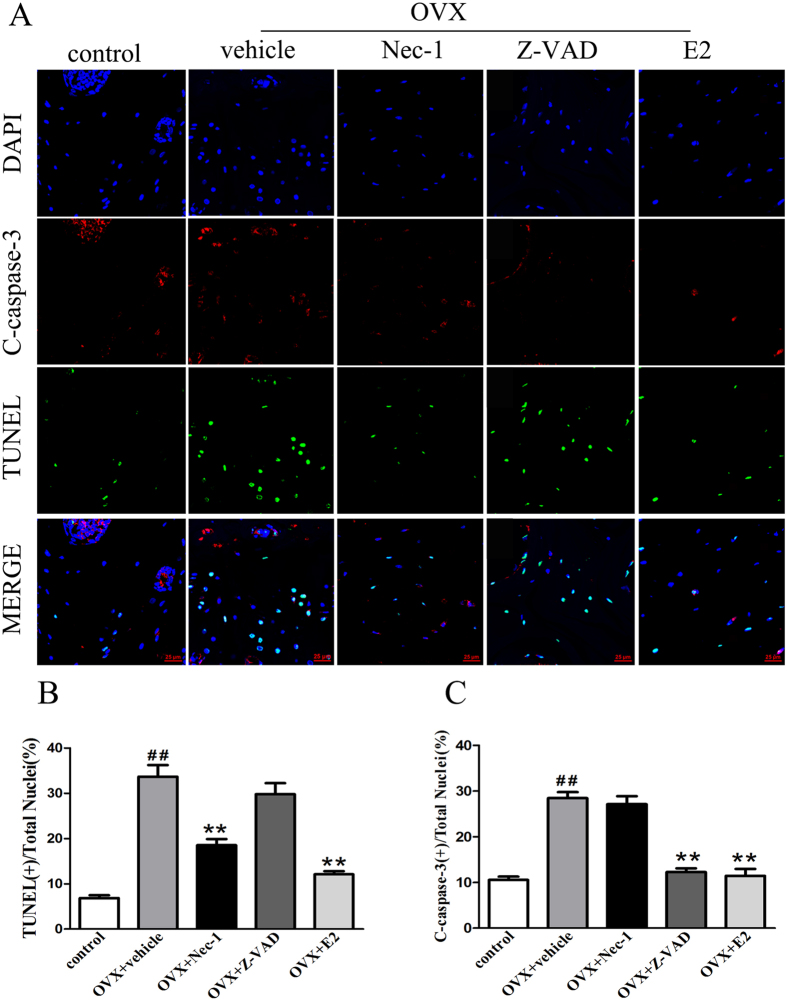
Detection of dead osteocytes using immunofluorescence of cleaved caspase-3 and *in situ* fluorescence TUNEL staining in the bone tissue of OVX rats. (**A**) TUNEL-stained (green fluorescence) cells in decalcified bone were co-stained to detect cleaved caspase-3 (red fluorescence) and the nucleus (DAPI, blue fluorescence). Scale bars represent 25 μm. (**B**) The data are presented as the % ratio of TUNEL-positive cells. (**C**) The data are presented as the % ratio of cleaved caspase-3-positive cells. The results are shown as the means ± S.E.M for three independent measurements. n = 6 rats per group, ^##^p < 0.01 compared with the control group, **p < 0.01 compared with the vehicle group. OVX: ovariectomized. C-caspase-3: cleaved caspase-3.

**Figure 3:**
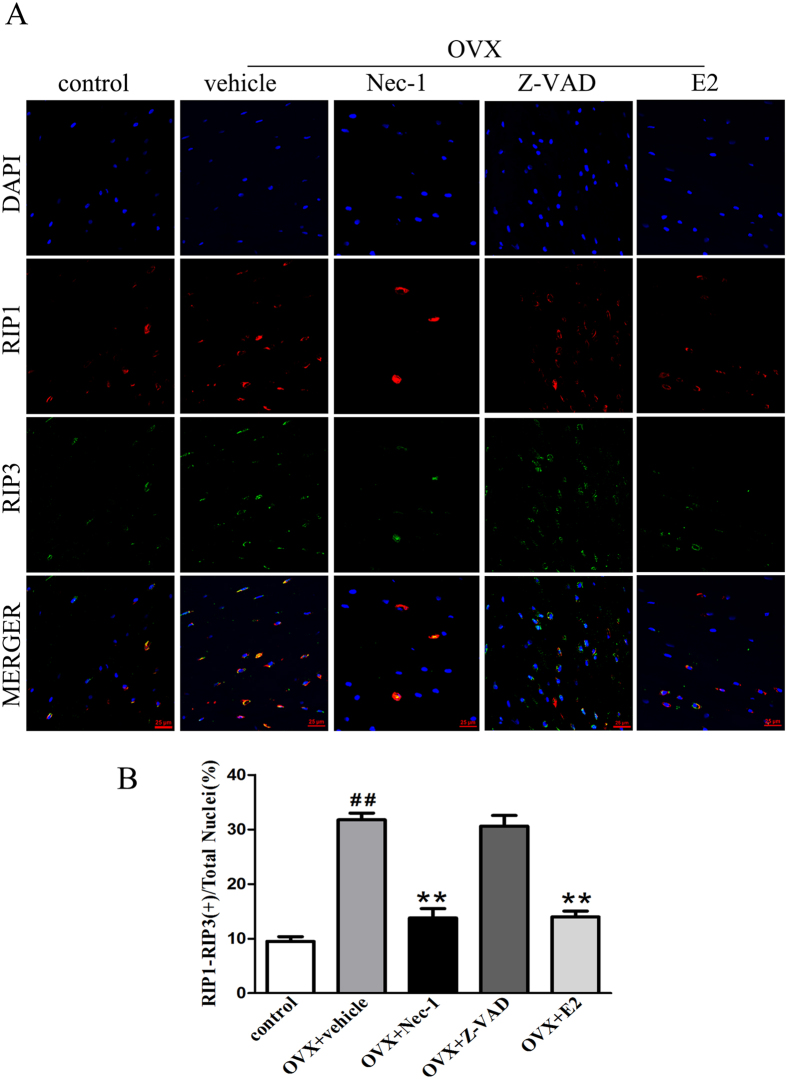
Detection of necroptotic steocytes using double immunofluorescence staining for RIP1 and RIP3 in the bone tissue of OVX rats. (**A**) Representative photomicrographs of decalcified bone sections stained to detect RIP1 (red fluorescence), RIP3 (green fluorescence), and the nucleus (DAPI, blue fluorescence). Scale bars represent 25 μm. (**B**) The data are presented as the % ratio of RIP1-RIP3 colocalization-positive cells. The results are shown as the means ± S.E.M for three independent measurements. n = 6 rats per group, ^##^p < 0.01 compared with the control group, **p < 0.01 compared with the vehicle group. OVX: ovariectomized.

Terminal deoxy nucleotidyl transferase dUTP nick end labeling (TUNEL) and anti-active caspase-3 staining are often used to determine the type of cell death that a cell undergoes. Typically, cells that are positive for TUNEL staining but negative for active caspase-3 are considered to be necrotic cells^18^,^19^,^20^. As shown in Fig. 2A, we found a group of osteocytes that were TUNEL-positive, but negative for cleaved caspase-3, suggesting that necroptosis is involved in osteocyte loss. In addition, we found that the percentage of TUNEL-positive osteocytes was significantly higher in the vehicle group than in the control group. There were significantly fewer TUNEL-positive osteocytes in the Nec-1 group than in the vehicle group (Fig. 2B). However, Nec-1 had no effect on the percentage of cleaved caspase-3-positive osteocytes (Fig. 2C). Treatment with Z-VAD significantly decreased the percentage of cleaved caspase-3-positive osteocytes (Fig. 2C). Compared with the vehicle group, E2 significantly decreased the percentage of cleaved caspase-3- and TUNEL-positive osteocytes (Fig. 2B,C). We also studied the localization of RIP1 and RIP3, which are critical biomarkers of necroptosis in osteocytes, via immunofluorescence. Using confocal microscopy, we observed that RIP1 colocalized with RIP3 in many of the osteocytes from OVX rats after 8 weeks, particularly in the cytoplasms of the osteocytes (Fig. 3A). Additionally, we found that the percentage of RIP1-RIP3 positive osteocytes was significantly higher in the vehicle group than in the control group. There were also significantly fewer RIP1-RIP3 positive osteocytes in the Nec-1 group than in the vehicle group. Nec-1 had a significant effect on the number of RIP1-RIP3-positive osteocytes induced by E2 deficiency (Fig. 3B).

**Figure 4:**
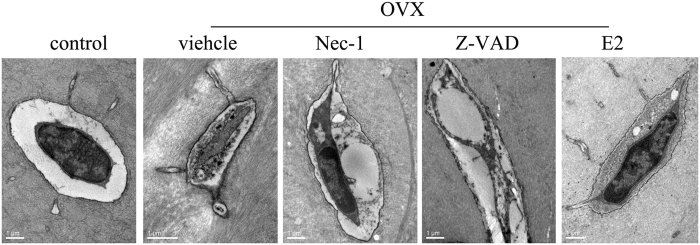
Changes to the osteocyte morphology in the bone tissue of OVX rats using TEM. Representative images of necroptotic osteocytes with typical necrotic morphological features were observed via TEM in the vehicle and Z-VAD groups. Scale bars represent 1 μm.

To better understand the morphological characteristics of the osteocytes in OVX rats, we performed transmission electron microscopy (TEM) to observe the ultrastructure of the osteocytes. Figure 4 shows the microstructural changes observed in osteocytes via TEM. In the vehicle group, we found an interesting phenomenon: many of the osteocytes displayed a necrotic morphology, appearing markedly swollen and showing membranolysis and organelle disappearance. These findings are consistent with the typical morphological features of necroptotic cell death^3^,^11^,^14^. In the group of OVX rats pretreated with Nec-1, the integrity of the osteocytes was better preserved than in the vehicle group. When the OVX rats were pretreated with E2, the cell morphology appeared almost normal. However, in the group of OVX rats pretreated with Z-VAD, we found that most of the osteocytes also displayed necrotic morphology.

These results suggest that osteocyte necroptosis may be inhibited in OVX rats pretreated with Nec-1.

### Effect of Nec-1 on biochemical bone markers in OVX rats

**Figure 5:**
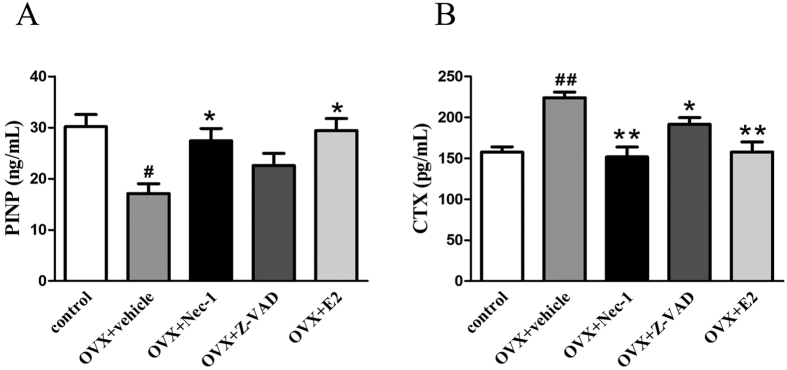
Effect of Nec-1 on biochemical bone markers in OVX rats. The levels of PINP and CTX in rat serum were analyzed using an ELISA. (**A**) PINP levels were increased in rats treated with Nec-1 or E2 compared with the vehicle group. (**B**) The levels of CTX were decreased in rats treated with Nec-1, Z-VAD or E2 compared with the vehicle group. Data are presented as the means ± S.E.M (n = 6 rats per group). ^#^p < 0.05 compared with the control group, ^##^p < 0.01 compared with the control group, *p < 0.05 compared with the vehicle group, **p < 0.01 compared with the vehicle group. OVX: variectomized.

To determine the effect of Nec-1 on biochemical bone markers in OVX rats, procollagen type I N-terminal propeptide (PINP) was examined as a marker of bone formation^21^, while the serum-localized C-terminal cross-linking telopeptide of type I collagen (CTX, a biochemical marker of bone resorption) was used as a bone resorption marker^22^. As shown in Fig. 5A,B, compared to the control group, the level of PINP was significantly decreased, while the level of CTX was increased in the vehicle group, suggesting that there was an inhibition of bone formation and stimulation of bone resorption in OVX rats after 8 weeks. Following E2 administration, the level of PINP was increased, while the level of CTX decreased. In addition, after necrostatin-1 administration, CTX expression was significantly decreased, while PINP expression dramatically increased. When pretreated with Z-VAD, PINP expression in OVX rats showed no significant difference from the OVX group that was not treated with Z-VAD, whereas the CTX level was decreased. These results suggest that E2 increases the rate of bone formation and decreases bone resorption. In addition, Nec-1 also appears to increase the rate of bone formation and to decrease bone resorption.

### Effect of Nec-1 on the microarchitecture of the bone in OVX rats

**Figure 6:**
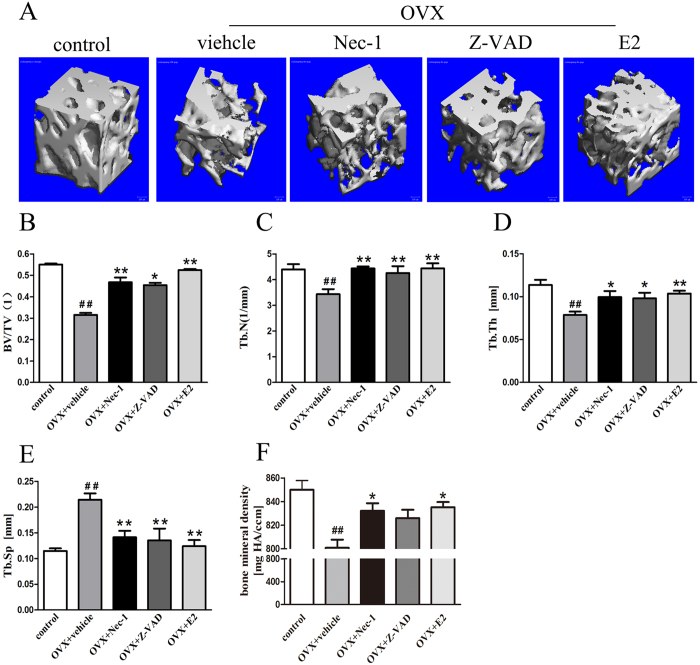
Effect of Nec-1 on the bone microarchitecturein OVX rats. Ward’s triangle was scanned using a micro-CT system. A series of planar transverse gray-value images were generated to reconstruct 3D images. The microarchitectural parameters, including BV/TV, Tb.Th, Tb.N, Tb.Sp, and BMD, were calculated automatically using the micro-CT system software. (**A**) Representative 3D-reconstructed images were obtained from rats from each treatment group. Scale bars represent 100 μm. Images of (**B**) BV/TV, (**C**) Tb.Th, (**D**) Tb.N, (**E**) Tb.Sp, and (**F**) BMD from various treatment groups are shown. Data are presented as the means ± S.E.M (n = 6 rats per group). ^##^p < 0.01 compared with the control group, *p < 0.05 compared with the vehicle group, **p < 0.01 compared with the vehicle group. BV/TV, bone volumefraction; Tb.Th, trabecular thickness; Tb.N, trabecular number; Tb.Sp, trabecular separation; BMD, bone mineral density; OVX: ovariectomized.

Next, bone microarchitecture parameters, including BV/TV (the bone volume over total volume), Tb.Th (trabecular thickness), Tb.N (trabecular number), Tb.Sp (trabecular separation) and BMD, were measured. The results are shown in Fig. 6. Eight weeks post-OVX, the levels of BV/TV (Fig. 6B), Tb.N (Fig. 6C) and Tb.Th (Fig. 6D) significantly decreased, while the Tb.Sp level (Fig. 6E) increased compared to the control group. These results suggest that E2 deficiency is involved in the reduction of bone mass. As shown in Fig. 6E, we also found that compared to the vehicle group, there was a statistically significant decrease in Tb.Sp after treatment with E2, while the other parameters were significantly increased. In the Nec-1 group pretreated with necrostatin-1, Tb.N, BV/TV and Tb.Th levels markedly increased, while the Tb.Sp level decreased. In addition, when rats were treated with Z-VAD, the levels of Tb.N, BV/TV and Tb were significantly increased, while the Tb.Sp level was decreased. These data suggest that Nec-1, Z-VAD and E2 are able to increase the trabecular bone volume.

Through micro-computed tomography (micro-CT) analysis, the BMD of the proximal femur was determined to be significantly reduced in the vehicle group compared to the control group. Upon treatment with E2, the BMD was increased (Fig. 6F). In addition, following necrostatin-1 administration, the BMD was significantly increased. When the OVX rats were treated with Z-VAD, there was no significant difference in the BMD between the Z-VAD and vehicle groups.

### Estrogen deficiency increases the production of TNF-α in bone marrow of OVX rats

**Figure 7:**
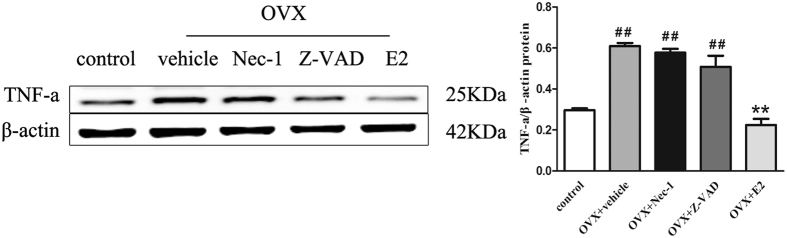
Western blotting analysis of TNF-α protein levels in the bone marrow of OVX rats. (**A**) Representative blots of TNF-α protein are shown. (**B**) Western blotting analyses for TNF-α protein expression in the bone marrow of rats in each treatment group are shown. The results are shown as the means ± S.E.M for three independent measurements. N = 6 rats per group, ^##^p < 0.01 compared with the control group, **p < 0.01 compared with the vehicle group. OVX: ovariectomized.

As TNF**-**α is produced mainly in hematopoietic precursors, we examined the expression of TNF-α by western blotting in each group bone marrow of rats. TNF-α protein expression was at low level in the control group, but increased sharply in the vehicle group (p < 0.01) (Fig. 7). Compared with the rats of the vehicle group, E2 significantly inhibited TNF-α protein expression (p < 0.01). Treatment with Z-VAD or Nec-1 had no effect on TNF-α protein expression.

### Nec-1 prevents TNF-α induced the mouse osteocyte cell line (MLO-Y4) necroptosis

**Figure 8:**
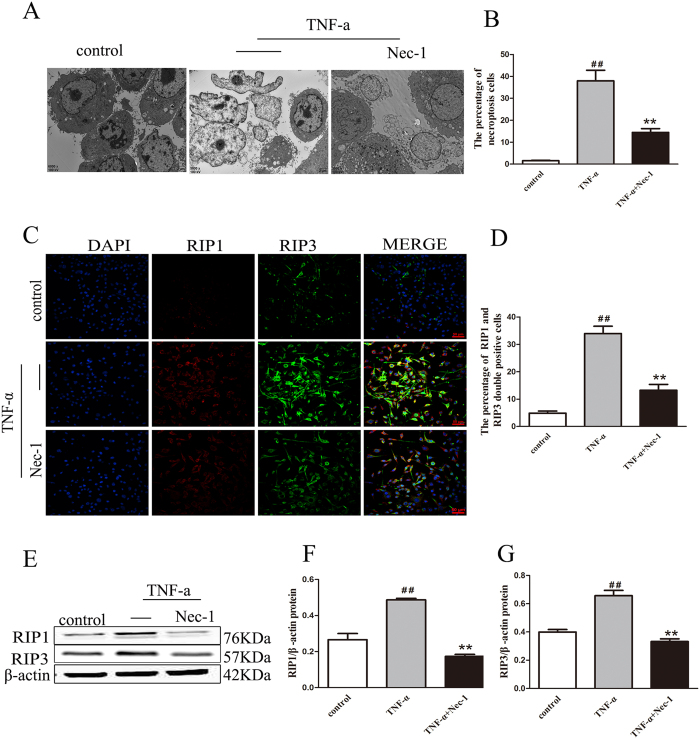
Effect of Nec-1 on MLO-Y4 necroptosis induced by TNF-α. (**A**) TEM photomicrographs of MLO-Y4 cells pretreated for 30 min with DMSO (1%), Nec-1 (30 mmol/L), subsequently treated with TNF-α (100 ng/mL) for 24 h. (Scale bar, 2 μm). (**B**) The data are presented as the percent ratio of MLO-Y4 cells necroptosis. (**C**) Immunofluorescence staining of RIP1 (red), RIP3 staining (green) and DAPI (blue) staining in MLO-Y4 cells pretreated for 30 min with DMSO (1%), Nec-1 (30 mmol/L), subsequently treated with TNF-α (100 ng/mL) for 24 h. (Scale bar, 50 μm). (**D**) The data are presented as the percent ratio of RIP1-RIP3 colocalization-positive cells. (**E**) Representative blots are shown. Western blotting analyses for RIP1 (**F**) and RIP3 (**G**) protein expression in MLO-Y4 in each treatment group are shown. The results are shown as the means ± S.E.M for three independent measurements. ^##^p < 0.01 compared with the control group, **p < 0.01 compared with the MLO-Y4 cells treated with only TNF-α.

The findings demonstrated that enhanced TNF-α is a key mechanism by which estrogen deficiency induces bone loss *in vivo*^23^. In current study, we report that estrogen deficiency increases expression of TNF**-**α in bone marrow in OVX rats, suggesting that abnormalities of TNF-α may relate to necroptotic osteocytes. So, MLO-Y4 cells were cultured *in vitro* to further investigate the mode of osteocyte death by TEM. Further results from cells experiments by TEM demonstrated that any of the MLO-Y4 stimulated with TNF-α displayed a necrotic morphology (Fig. 8A). More importantly, pretreatment with TNF-a plus Nec-1, necroptotic incidence of MLO-Y4 sharply reduced (Fig. 8B).

We studied the expression of RIP1 and RIP3 in MLO-Y4, via immunofluorescence. Using confocal microscopy, we observed that RIP1 colocalized with RIP3 in many of the MLO-Y4 treated with TNF-a (100 ng/ml) for 24 h, particularly in the cytoplasms of the osteocytes (Fig. 8C). The percentage of RIP1-RIP3 positive MLO-Y4 was significantly higher in MLO-Y4 stimulated with TNF-α than in the control group (p < 0.01) (Fig. 8D). Importantly, stimulation with TNF-α combined with Nec-1 significantly decreased RIP1-RIP3 positive MLO-Y4 (p < 0.01) (Fig. 8D).

We also investigate the effects of TNF-α on the expression of RIP1 and RIP3 in MLO-Y4 by western blotting (Fig. 8E). Interestingly, the expression of RIP1 and RIP3 were significantly higher in MLO-Y4 cells stimulated with TNF-α than cells stimulated with PBS alone (p < 0.01) (Fig. 8F,G). Stimulation with TNF-α combined with Nec-1 significantly inhibited RIP1 expression (p < 0.01) (Fig. 8F) but had no effect on RIP3 (Fig. 8G).

These results *in vitro* were consistent with results from the OVX rats *in vivo*, suggesting that MLO-Y4 necroptosis stimulated with TNF-α may be inhibited pretreated with Nec-1.

## Discussion

With an aging population, the number of postmenopausal osteoporosis cases will continue to steadily increase. In the past decade, it has been confirmed that osteocyte apoptosis is an important pathological phenomenon involved in E2 deficiency-induced osteoporosis^24^,^25^,^26^. In addition to apoptosis, our previous study showed that osteocyte necroptosis is also responsible for osteocyte loss in OVX rats^3^. In the present study, we found that Nec-1 treatment effectively suppresses osteocyte necroptosis and thereby inhibits osteoporosis progression in OVX rats.

The complete mechanism underlying necroptosis is still unclear, but RIP1/3 have been shown to play critical roles in the necroptotic pathway^7^,^8^,^9^. The expression of RIP3 and the RIP1-RIP3 binding complex is a prerequisite for RIP1 activation and leads to necroptosis^8^,^9^,^16^. In our study, RIP1 and RIP3 expression levels were measured in bone tissue via western blot analysis, and RIP1/RIP3 colocalization assays were also performed. We found that both RIP1 and RIP3 expression levels were significantly higher in the vehicle group than in the control group 8 weeks post-surgery. We also observed that many of the osteocytes from rats in the vehicle group had morphological changes that suggested necrosis. Furthermore, the percentage of RIP1-RIP3 positive osteocytes was significantly higher in the vehicle group than in the control group. We also found that MLKL expression in the bone tissue was increased, and Nec-1 reduced its expression levels. This result is largely consistent with the changes in the expression levels of RIP1 and RIP3 and suggests that MLKL, a downstream signaling molecule of RIP1 and RIP3, may also participate in signaling pathways that promote osteocyte necroptosis. Together, these results suggest that necroptosis contributes to bone loss in OVX rats. One of the most important features of necroptosis is that it can be specifically inhibited by a small molecule, Nec-1, which targets the death domain of the RIP1 kinase but cannot be blocked by an inhibitor of apoptosis, such as Z-VAD^27^. We found that Nec-1 treatment significantly reduced RIP1 over expression in OVX rats, but the increased expression could not be suppressed by Z-VAD treatment. This result is in agreement with the above features of necroptosis and further confirms that necroptosis is involved in osteocyte loss in OVX rats. Apoptosis is a specific type of programmed cell death that is coordinated by members of the caspase family of cysteine proteases. Caspase-3 in particular plays a central role in the execution phase of cell apoptosis. We found that cleaved caspase-3 expression was significantly higher in the vehicle group than in the control group. These results suggest that apoptosis and necroptosis may occur simultaneously in the osteocytes of OVX rats.

RIP1 is an adaptor kinase that acts downstream of death domain receptors and is crucial for necroptosis^28^. Necrostatin-1 specifically targets the RIP1 kinase and can thereby block the formation of the necrosome, which consists of a complex of the RIP1 and RIP3 proteins. Importantly, it has been previously confirmed that Nec-1 has no effect on apoptosis^29^. In this study, we found that Nec-1 treatment effectively suppressed RIP1 and MLKL expression in OVX rats. We further found that the expression of RIP1-RIP3-positive osteocytes were reduced following Nec-1 treatment. TUNEL staining has long been considered the gold standard in apoptosis assays. However, Grasl-kraupp B *et al*. reported that necrosis can also generate DNA fragments that react with the TUNEL reaction solution^12^,^30^. Therefore, TUNEL-positive cells might not necessarily be indicative of apoptosis. We found that TUNEL-positive osteocytes were significantly decreased after treatment with Nec-1. Based on our TEM results, the necrotic features of osteocytes induced by E2 deficiency were markedly attenuated by Nec-1 treatment. Our results suggest that Nec-1 effectively prevents osteocyte necroptosis in OVX rats.

Apoptosis has been suggested to play a critical role in the pathogenesis of E2 deficiency-induced osteoporosis^31^,^32^. In the present study, the cleaved caspase-3 expression level was enhanced in bone tissue from OVX rats, an effect that was inhibited by Z-VAD. These findings were obtained using western blotting analysis. Interestingly, we observed few apoptotic osteocytes in the TEM images of OVX rats. A possible explanation for this finding is that few osteocytes undergo apoptosis in the stages that we evaluated, likely because osteocyte apoptosis is an early event in E2 deficiency-mediated bone loss. Many previous studies have demonstrated that increased osteocyte apoptosis occurs within 4 weeks of E2 withdrawal^28^,^29^. Therefore, we can speculate that necroptosis is the primary mechanism mediating osteocyte death in OVX rats 8 weeks post-surgery. Galluzzi *et al*. suggested that apoptosis and necroptosis can occur simultaneously in cells and that necroptosis could even switch to apoptosis under special conditions^33^. However, we did not find any evidence to suggest that osteocyte apoptosis and necroptosis occurred simultaneously in osteocytes in OVX rats. Therefore, the relationship between osteocyte apoptosis and necroptosis is not yet clear in OVX rats.

Changes to the microarchitecture of trabecular bone can be used to evaluate bone quality^34^, and biochemical markers have been employed to reflect bone remodeling^35^. In the present study, we found that E2 deficiency markedly decreased PINP serum levels in rats, while it increased CTX expression. The OVX rats were also found have a high level of Tb.Sp and decreased levels of BV/TV,Tb.N and Tb.Th. These findings are consistent with those of previous studies^36^. These changes suggest that there is a decline in bone formation and an activation of bone resorption in OVX rats. However, E2 replacement reduced bone loss in the OVX femur at 8 weeks post-surgery. Quantitative micro-CT data indicated that there was a significant difference in the levels of BV/TV and other parameters at 8 weeks post-surgery between the vehicle group and the Nec-1 group. Nec-1 pretreatment increased the levels of BV/TV, Tb.N and Tb.Th in OVX rats, which may indirectly lead to adecrease in Tb.Sp expression. This finding was surprising, considering the capacity of Nec-1 administration to prevent OVX-induced osteoporosis. We also found that Nec-1 significantly increased the serum levels of PINP and decreased the serum levels of CTX. This finding suggests that necrostatin-1 may accumulate during bone formation in OVX rats.

Despite many studies on TNF-family ligands and receptors in bone, the detailed mechanism of the functional TNF-α in osteocytes remains to be elucidated. In the present study, we investigated the effects of TNF-α on osteocytes *in vivo* and *in vitro*, and demonstrated that TNF-α can induce osteocytes necroptosis and this effect was reversed pretreated with Nec-1. All of these data suggested that increasing TNF-α could induce osteocytes necroptosis under conditions of estrogen deficiency.

In summary, RIP1- and RIP3-mediated necroptosis is a redundant mechanism of osteocyte death in addition to apoptosis, and Nec-1 can protect against bone loss in the context of E2 deficiency and may constitute a novel therapeutic strategy for osteoporosis treatment. These findings may have potential clinical applications, given that our rodent model mimics the clinical conditions of E2-deficient women.

## Materials and Methods

### Experimental design

#### Animals

Sixteen-week-old healthy female Sprague-Dawley rats (from the animal laboratory center of Chongqing Medical University) were used in this study. All of the rats were housed in cages under standard laboratory conditions with a 12-h dark/12-h light cycle and had free access to water and a standard rodent diet. After a 7-day adaptation period, sixty rats were randomly assigned to either the OVX group or the control group. Forty-eight animals underwent bilateral OVX under pentobarbital sodium anesthesia. The remaining 12 animals underwent sham surgery, in which the ovaries were exteriorized and then replaced in the abdominal cavity. The research was approved by the ethical committees of the First Affiliated Hospital of Chongqing Medical University. The methods were carried out in accordance with the approved guidelines. The study complied with the Declaration of Helsinki.

### Drug administration

Necrostatin-1 (Nec-1, Sigma-Aldrich, USA), Z-VAD-fmk (Z-VAD, MP Biomedicals, Solon, OH, USA) and 17β-estradiol (E2, Sigma Chemical Co. USA) were dissolved in 10% dimethyl sulfoxide (DMSO, Sigma, USA). Rats underwent the OVX surgery, recovered for 4 weeks, and were then randomly assigned to one of four groups (n = 12 per group): the Nec-1 group, the Z-VAD group, the E2 group and the vehicle group. Rats were injected intraperitoneally with Nec-1 (1.65 mg/kg/d^37^), Z-VAD (1.0 mg/kg/d^38^), E2(10 μg/kg/day^37^) or an equal volume of 10% DMSO (vehicle) and were then injected once per day for 4 additional weeks. The control group was administered a vehicle solution. At 8 weeks post-surgery, the rats were sacrificed. This time point was chosen because osteocyte death peaks at 8 weeks after OVX^3^.

### Sample harvest and preparation

The animals were weighed at the beginning and end of the experiment. Animals were anesthetized as described above, and blood samples were collected by cardiac puncture. Next, the animals were euthanized by cervical dislocation. The blood samples were centrifuged to separate the plasma. Serum was stored at −80 °C for subsequent biochemical measurements. After the animals were sacrificed, both the right and left proximal femurs (5 mm distal and proximal to Ward’s triangle) were dissected, and the soft tissue was removed from the bones. The bone marrow was removed from the proximal femur through repeated washes with PBS. The right femurs of rats from each group were briefly immersed in liquid nitrogen for subsequent western blot analyses (n = 6 rats/group). Six of the left femurs from each group were fixed in 10% neutral buffered formalin for 48 hours at 4 °C for micro-CT analysis. Following micro-CT measurements, the left femurs were decalcified in 15% ethylenediaminetetraacetic acid (EDTA, pH7.4) for 4 weeks for immunofluorescence analysis. The other six left femurs were fixed in 2.5% glutaraldehyde for 24 hours at 4 °C and were then decalcified in 15% EDTA (pH 7.4) for 4 weeks prior to TEM analysis.

### Western blotting

The proximal femur specimens that were frozen in liquid nitrogen were placed in a mortar for pulverization, followed by homogenization in ice-cold radioimmunoprecipitation assay (RIPA) buffer (Beyotime, Nantong, Jiangsu, China). The total protein concentration was determined using a Bicinchoninic Acid (BCA) Protein Assay kit (Beyotime, Nantong, Jiangsu, China), according to the manufacturer’s instructions. Samples containing approximately 80 μg of protein were separated via SDS-PAGE, and the proteins were transferred to a polyvinylidene difluoride (PVDF) membrane (EMD Millipore, Billerica, Massachusetts, USA). After blocking non-specific binding sites with a 5% non-fat dry milk solution for 1.5 hours at room temperature, the membranes were incubated overnight at 4 °C with the following primary antibodies:anti-RIP1 monoclonal antibody (Abcam, Cambridge, MA, USA), anti-RIP3 polyclonal antibody (Abcam, Cambridge, MA, USA), anti-MLKL polyclonal antibody (Santa Cruz Biotechnology, CA, USA), anti-cleaved caspase-3 monoclonal antibody (Cell Signaling Technologies, Danvers, MA, USA), anti-TNF-α monoclonal antibody (Cell Signaling Technologies, Danvers, MA, USA) and anti-β-actin monoclonal antibody (Santa Cruz Biotechnology, CA, USA). Western blotting was performed using conventional methods, as described previously^39^. The protein bands of interest were visualized using an ECL detection kit (Beyotime, Nantong, Jiangsu, China). Protein expression was quantified via densitometry analysis using Image Lab version 2.1 (Bio-Rad). The quantitative densitometric values for each protein were normalized to β-actin.

### Immunofluorescence detection of cleaved caspase-3 and *in situ* fluorescence TUNEL staining

The proximal femur samples were fixed in 10% neutral buffered formalin for 48 hours at 4 °C and were then decalcified in 15% EDTA at room temperature for 4 weeks. Sagittal tissue sections (4 μm thick) were prepared for immunofluorescence and *in situ* fluorescent TUNEL staining. After three washes with 0.1 MPBS, the sections were incubated with 20 μg/ml proteinase K for 15 minutes at 37 °C. After treatment with 0.1% Triton X-100, the sections were blocked with 10% goat serum, incubated with a rabbit polyclonal antibody against cleaved caspase-3 (Cell Signaling Technologies, Danvers, MA, USA; 1:100 dilution) overnight at 4 °C, and then incubated with Dylight 594 AffiniPure goat anti-mouse IgG (EarthOx, San Francisco, USA). After three washes with 0.1 MPBS, the sections were assayed using an *in situ* cell death detection kit (Roche, Basel, Switzerland), according to the manufacturer’s instructions. All of the sections were counterstained with 4′,6-diamidino-2-phenylindole (DAPI). Finally, the numbers of total cells and TUNEL-positive cells, with and without cleaved caspase-3-positive cells, were counted in three to five non-contiguous high-power fields for each specimen using a laser scanning confocal microscope (LEICA TCS SP2, Wetzlar, Germany). The percentages and numbers of TUNEL-positive cells and cleaved caspase-3-positive cells were calculated with and without these values. The cell counting was performed by a pathologist blinded to the experimental conditions.

### Double immunofluorescence staining for RIP1 and RIP3

Tissue sections were treated as described above. After treatment with 0.1% Triton X-100, the sections were blocked with 10% goat serum and incubated over night at 4 °C with a mouse RIP1 monoclonal antibody (Abcam, Cambridge, MA, USA; 1:100 dilution) and a rabbit RIP3 polyclonal antibody (Abcam, Cambridge, MA, USA; 1:100 dilution), followed by incubation with Dylight 594 AffiniPure goat anti-mouse IgG and Dylight 488 AffiniPure goat anti-rabbit IgG (EarthOx, San Francisco, CA, USA). After three washes with 0.1 MPBS, the sections were counterstained with DAPI. Finally, the numbers of total cells and RIP1/-RIP3-positive cells were determined using a laser scanning confocal microscope (LEICA TCS SP2, Wetzlar, Germany).

### Transmission electron microscopy

Specimens from the Ward’s triangle area (1 mm^3^) were fixed in 2.5% glutaraldehyde for 24 hours at 4 °C and then washed three times with 0.1 M PBS (pH 7.4). Next, the specimens were demineralized in 15% EDTA for 2 weeks at room temperature. After three washes with 0.1 M PBS, the tissue fragments were fixed in 2% osmium tetroxide for 1 hour and then block-stained with 2% uranyl acetate. The bone tissue was then embedded in epoxy resin and dehydrated. Tissue sections (80 nm thick) were cut and stained with uranyl acetate and lead citrate. The ultrastructure of the bone tissue was observed via TEM (Hitachi-7500, Hitachi, Tokyo, Japan).

### Measurements of collagen type I cross-linked C-telopeptide (CTX) and procollagen type I N-terminal propeptide (PINP)

An enzyme-linked immunosorbent assay (ELISA) was performed to measure the levels of CTX (USCN LIFE, Wuhan EIAab Science Co, Ltd, Wuhan, China) and PINP (Elabscience Biotechnology, Wuhan Elabscience Biotechnology Co. Ltd, Wuhan, China) in rat serum, according to the manufacturer’s protocol.

### Micro-CT measurements

Ward’s triangle is a region in the intersection between the compressive and tensile trabecular systems, and it is the concentrated stress site for the cancellous bone within the femoral neck region and a key area of osteoporosis in the femoral neck^40^. To analyze the 3D microarchitecture of the bone, Ward’s triangle was scanned with a micro-CT system (viva CT40, SCANCO Medical AG, Zürich, Switzerland). The volume of interest(VOI, 29 × 29 × 29 μm^3^) was selected using a semiautomatic contouring method. The scans were performed with the following settings: 15 μm resolution, 70 kVp, 114 μA, and 250 ms integration time. A series of planar transverse gray-value images was generated to reconstruct 3D images. The microarchitectural parameters within the VOI, including BMD, BV/TV, Tb.Th, Tb.N, and Tb.Sp, were calculated automatically using the micro-CT system software.

### Cell culture and experiment

Murine long bone osteocyte Y4 (MLO-Y4) was purchased from the Cell Bank of the Chinese Academy of Sciences (Beijing, China). MLO-Y4 were cultured in DMEM/F12 culture medium (Gibco Life Technologies, Carlsbad, CA, USA) supplemented with 10% fetal bovine serum (Gibco Life Technologies, Carlsbad, CA, USA). All cells were cultured in a humidified incubator containing 5% CO2 at 37 °C. MLO-Y4 were plated at low density (2.5 × 10^4^ cells) in triplicate in 12-well plates in DMEM/F12 culture medium. The synchronized MLO-Y4, cultured in serum-free medium for 24 h before each experiment, were pretreated for 30 min at 37 °C with DOSM (1%) or Nec-1 (30 mmol/l) and then treated with TNF-α (100 ng/ml) for 24 h^37^,^41^. Following treatment, Cells (5 × 10^6^) were harvested, and prefixed with 2.5% glutaraldehyde for 2 h. these cells were made using a transmission electron microscope (Hitachi-7500, Japan) as described previously. The slides of cells were harvested, air-dried, and fixed in 10% neutral buffered formalin solution (Sigma, USA) for 10 min at room temperature. After rinsing in 0.1M PBS (pH 7.4) containing 0.3% Triton X-100 (PBST), the cells were applied for immunofluorescent staining. Cells (5 × 10^8^) were harvested and lysed in ice-cold RIPA buffer (Beyotime, Jiangsu, China). Then, the total protein concentration was determined using an Enhanced BCA Protein Assay kit (Beyotime, Jiangsu, China) according to the manufacturer’s instructions. Protein samples were used to analysis expression of RIP1 and RIP3.

### Statistical analysis

Data are presented as the means ± standard errors of the mean (SEM). Statistical analyses were performed with SPSS software (version 17.0). Significant differences between treatment groups were evaluated using an ANOVA followed by a least significant difference (LSD) or Dunnett’s post-hoc test. P < 0.05 was considered statistically significant.

## Additional Information

**How to cite this article**: Cui, H. *et al*. Necrostatin-1 treatment inhibits osteocyte necroptosis and trabecular deterioration in ovariectomized rats. *Sci. Rep.* **6**, 33803; doi: 10.1038/srep33803 (2016).
